# Concordance of movements and songs enhances receiver responses to multimodal display in the starling

**DOI:** 10.1038/s41598-024-54024-w

**Published:** 2024-02-13

**Authors:** Stanisław Rusiecki, Paweł Ręk

**Affiliations:** Department of Behavioural Ecology, Faculty of Biology, Institute of Environmental Biology, Adam Mickiewicz University, Poznań, Poland

**Keywords:** Behavioural ecology, Perception, Sensory processing, Social behaviour

## Abstract

Many animals produce signals that consist of vocalizations and movements to attract mates or deter rivals. We usually consider them as components of a single multimodal signal because they are temporally coordinated. Sometimes, however, this relationship takes on a more complex spatiotemporal character, resembling choreographed music. Timing is important for audio-visual integration, but choreographic concordance requires even more skill and competence from the signaller. Concordance should therefore have a strong impact on receivers; however, little is known about its role in audio-visual perception during natural interactions. We studied the effects of movement and song type concordance in audio-visual displays of the starling, *Sturnus vulgaris*. Starlings produce two types of movements that naturally appear in specific phrases of songs with a similar temporal structure and amplitude. In an experiment with a taxidermic robotic model, males responded more to concordant audio-visual displays, which are also naturally preferred, than to discordant displays. In contrast, the effect of concordance was independent of the specific combination of movement and song types in a display. Our results indicate that the concordance of movements and songs was critical to the efficacy of the display and suggest that the information that birds gained from concordance could not be obtained by adding information from movements and songs.

## Introduction

Animal acoustic signals are often accompanied by body movements. In its simplest form, this relationship can be mechanistic, such that movement itself produces sound. For example, birds, bats, and insects produce sonations using movements of their limbs or wings^1^. Body movements can also accompany sound signals more accidentally. Such an interaction takes place, for example, during migration, when individuals call to each other to maintain contact^2,3^. Often, however, body movements and sounds are components of complex multimodal displays. Then, both modalities have signalling functions and are functionally related. For example, male lance-tailed manakins (*Chiroxiphia lanceolata*) court females by combining vocalizations with dance movements^4^, while male little brown frogs (*Micrixalus saxicola*) call and perform foot flagging displays in male–male agonistic interactions^5^. In some cases, such as in duetting species, the functional relationship between movements and songs is so strong that independent components are very rare, even if they can be produced separately^6^.

In audio-visual signals, body movements influence responses to songs, and this effect may take many forms. When visual and acoustic components of multimodal signals produce qualitatively similar effects separately, their joint effect can be characterized by an additive or even superadditive intensity of the reaction. European robins (*Erithacus rubecula*), for example, respond more to red-breasted singing models than to brown-breasted singing models or red-breasted quiet models^7^. Similarly, Eastern gray squirrels (*Sciurus carolinensis*) react more strongly to alarm signals consisting of the bark and tail flag than to independent components^8^. Sometimes isolated movements are rare and do not elicit responses themselves but affect responses when interacting with singing. Male blue‒black grassquits (*Volatinia jacaranda*), for example, leap out of the grass to increase the detectability of the call^9^. Movements may thus work as amplifiers or attention grabbers to increase detection or discrimination of the acoustic component^10^. Furthermore, movements can qualitatively modulate the function of the acoustic signals. In duetting magpie-larks (*Grallina cyanoleuca*), for example, the syndrome of correlated territorial behaviours in response to the intruder's songs differs from the syndrome in response to the songs accompanied by movements^6^. Finally, singing in interaction with movement can elicit a different response than singing itself or elicit a response only in interaction with the movement. For example, in Túngara frogs, only audio-visual signals of males attract females—isolated components are ignored^11^.

In addition to the sounds and movements themselves, the responses to the audio-visual signal may depend on the specific match of components. In Túngara frogs, the common effect of the movements of the resonator sac and respective calls weakens with a decrease in temporal coordination^12^, which suggests that the timing of both components is crucial for their integration. A similar effect of audio-visual uncoordination was observed in magpie-lark duetting displays^13^, but in these birds, movement is not necessary to elicit a response^6^. This therefore suggests that a lack of temporal coordination can sometimes have even worse consequences for the efficacy of a multimodal signal than the lack of one component. Furthermore, in some species, responses to the audio-visual signal may depend on matching certain types of movement and sound. For example, in the superb lyrebird (*Menura novaehollandiae*), song types match specific dance movements to create potent mating displays^14^. Chimpanzees, in turn, can combine various vocalizations with different gestures that together prompt different reactions in receivers^15^. Finally, in some species, such as Montezuma oropendola (*Psarocolius montezuma*), individuals adjust acoustic and visual features of their display in a way that the loudest part of their song is matched with the high range acrobatic movements and more quiet songs with less distinct body movements^16^. In this case, the timing is coarse, and the match concerns the amplitude of calls and range of motion, not at the fine scale of following notes but at the entire display. This concordance is therefore not mechanistic, but it is also not accidental. Nevertheless, apart from early research into the role of auditory target loudness and visual attractor size in human multimodal perception^17,18^, little is known about the role of such choreographic concordance in communication.

We studied the relationship between sounds and movements in multimodal displays of the starling (*Sturnus vulgaris*). Starling is a medium-sized hole-nesting passerine characterized by high sociality and a complex reproductive system^19^. In groups, males engage in numerous interactions, establishing specific dominance hierarchies that operate in roosting and breeding areas, and males are often polygynous, defending additional breeding sites^20,21^. Usually, males compete with their displays, countersinging and flapping their wings intensely^22–24^. Males perform two distinct types of movements while singing their elaborated songs. Both types of movements are tightly linked with the song, only exceptionally being produced without a sound^19,22^. At the same time, each type of movement usually accompanies a specific, structurally concordant song type; expressive wing-waving is combined mainly with conspicuous high-frequency phrases, whereas indistinct wing-flicks are typically associated with quiet rattle songs^22^. Due to the complexity of starling songs, such matching requires skill and competence on the part of the sender. Therefore, a correct match should give the sender an advantage in interactions with rivals or at least improve its perceived quality. If songs and wing movements arranged concordantly enhance the efficacy of the display, then the concordance should potentially be of great importance in the complex social interactions of starlings and translate into the fitness of the signaller.

We used the robotic bird model and acoustic playback to experimentally test whether the natural concordance of movement and song types affects the efficacy of audio-visual signals. We compared the responses of adult starlings to audio-visual displays consisting of concordant movements and songs (Waving + High-frequency and Flick + Rattle) and discordant movements and songs (Waving + Rattle and Flick + High-frequency) to test two predictions. First, we predicted that starlings should react more to concordant than to discordant signals. Second, we predicted that as long as the display is concordant or discordant, the responses should be independent of the specific combination of movement and song type.

## Results

**Figure 1:**
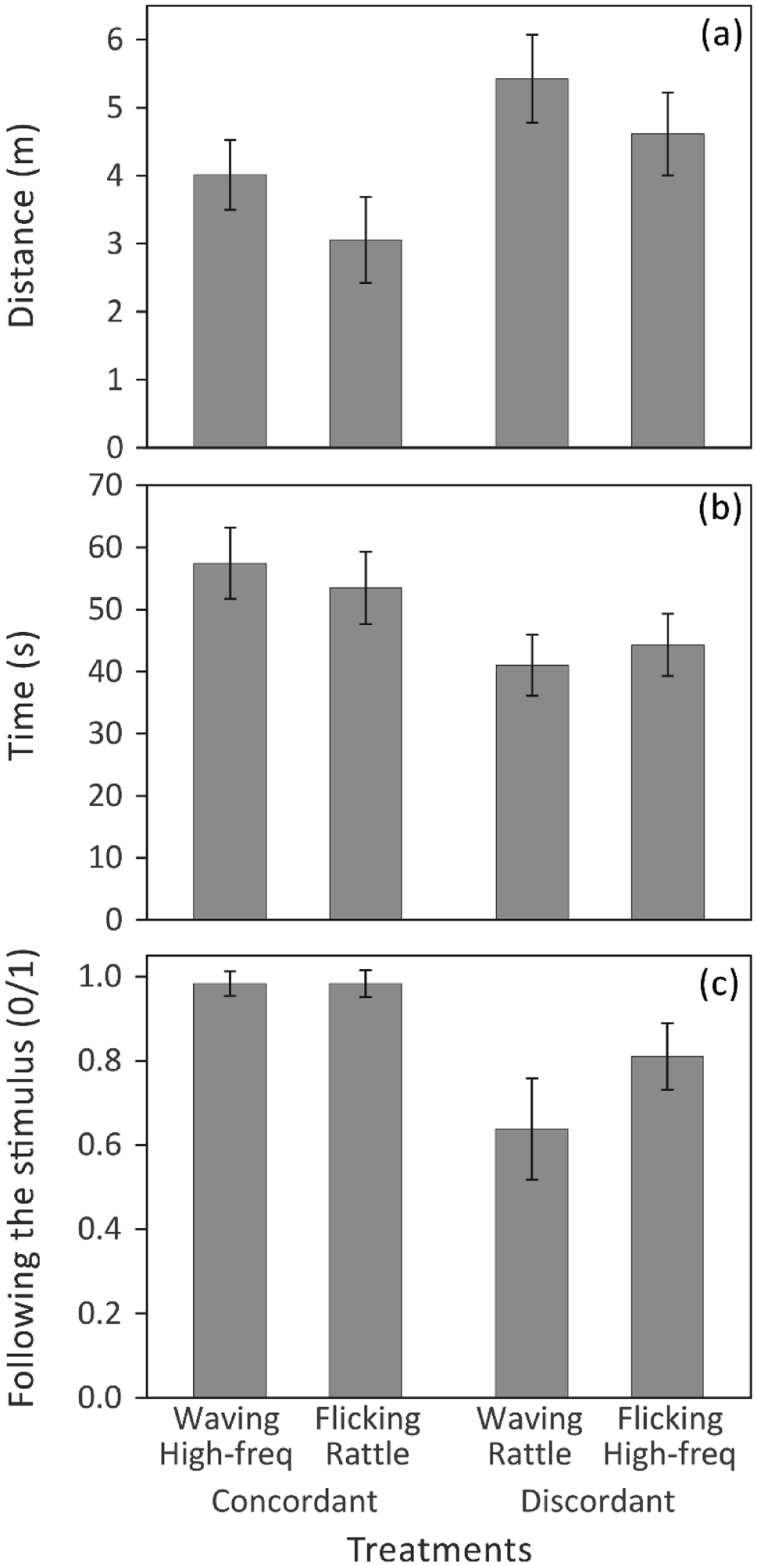
Response of wild starlings to four combinations of movement and song types displayed by the robotic model. Shown are responses to high-frequency and rattle song phrases accompanied by wing waving or flicking movements for 3 different response variables: (**a**) the minimum distance to the taxidermic model (**b**), time spent by male starlings near the taxidermic model, and (**c**) the following of the audio-visual stimulus. Boxes show means ± se.

Starlings responded to the concordance of movement and song types. Overall, there was an effect of playback treatment on all measures of response (Fig. 1, Table 1). This effect, however, resulted mainly from a stronger reaction of birds to concordant than to discordant stimuli (Fig. 1, Table 1) and not to differences between both concordant treatments (Waving + High-frequency vs. Flick + Rattle) or between both discordant treatments (Waving + Rattle vs. Flick + High-frequency) (Fig. 1; treatment nested in concordance—Table 1). Furthermore, pairwise comparisons of responses to treatment were significant only when we compared concordant versus discordant displays (Table 2), and we did not find any significant differences between the responses to two concordant treatments or two discordant treatments (Table 2).

**Table 1:** The effect of treatment and audio-visual concordance on three response variables.

Effect (df)	Distance		Time		Following the stimulus	
	F	P	F	P	F	P
Intercept (3, 70) * Treatment (3, 70)	2.89 2.89	0.042 0.042	3.42 3.42	0.022 0.022	3.59 3.59	0.018 0.018
Intercept (3, 70) ** Concordance (1, 70) Treatment (Concordance) (2, 70)	2.89 6.08 1.59	0.042 0.016 0.211	3.42 8.50 0.50	0.022 0.005 0.607	3.59 7.42 1.49	0.018 0.008 0.232

MIXED models with either treatment effect * or concordance and treatment nested in concordance effects **.

**Table 2:** Pairwise comparisons between treatment means (Fisher’s LSD test).

Treatments compared		Response variables – P		
		Distance	Time	Following the stimulus
*Waving* + *High-frequency*	*Flicking* + *Rattle*	0.182	0.411	1
*Waving* + *High-frequency*	Waving + Rattle	0.064	**0.005**	**0.003**
*Waving* + *High-frequency*	Flicking + High-frequency	0.572	0.027	0.226
*Flicking* + *Rattle*	Waving + Rattle	**0.004**	0.062	**0.003**
*Flicking* + *Rattle*	Flicking + High-frequency	0.074	0.187	0.231
Waving + Rattle	Flicking + High-frequency	0.214	0.570	0.094

Concordant treatments are italicised.

Significant values are in [bold].

## Discussion

Our experiment showed that displays with concordant movements and songs prompted stronger responses than discordant displays. Starlings stayed longer near the robotic model, approached the model closer, and were more likely to follow the model when the movements matched songs as in natural displays. These results indicate that starlings were able to distinguish natural pairs of songs and movements from unnatural pairs. In contrast, the concordance effect was independent of the specific combination of movements and songs in the audio-visual display. These results suggest, therefore, that the display components themselves were less important for receivers than how the two modalities were juxtaposed with each other. Overall, we suggest that the positive effect of concordance in the starling audio-visual displays is a consequence of the fact that both components together represent a multimodal percept^25^. In other words, the receivers obtain some information only if they have access to a specific combination of both components.

Birds responded more strongly to the typical combinations of movements and songs than to the atypical combinations. Starlings most often produce both modalities in specific combinations, but this is not obligatory, as each type of movement can occur in any phrase of the song and independently^19,23^. Therefore, there are probably no anatomical or physiological constraints on the sender's side to produce specific combinations. Nevertheless, even if such a constraint existed, it would not clearly explain the reactions of receivers. In fact, the observed effect on the receiver's side may be due to the negative impact of discordant stimuli on perception. Many studies have shown that the discrepancy between sensory modalities leads to crossmodal conflicts and depression of responses^26^. Considering our results, we think that both interpretations may be correct—in a concordant display, wing movements might enhance song efficacy, while in a discordant display, movements might hamper acoustic perception.

Our results suggest that the effect of concordance was independent of the specific combination of movement and song types in a display. Although both concordant treatments (Waving + High-frequency and Flicking + Rattle) and both discordant treatments (Waving + Rattle and Flicking + High-frequency) differed simultaneously in movement and song type, they prompted similar responses. This suggests that as long as the movement and song were concordant or discordant within a single display, it no longer mattered which particular pair of components produced the effect. In contrast, concordant and discordant stimuli always had one component in common, yet responses to them differed significantly. The fact that the difference of one component produced a stronger effect than the difference of two components also indicates that the concordance is not based on the selection of specific components for the display but on the selection of both components of the display at once based on their natural pairing. It is important to emphasize here that our experiment did not test the effect of isolated songs or movements but the effect of specific combinations of modalities. To achieve this, we standardized the intensity of both song types. However, under natural circumstances, high-frequency phrases are produced with a higher intensity than rattle phrases^27^. Hence, we cannot exclude that in real interactions, displays with high-frequency phrases might elicit stronger responses, both in concordant and discordant displays. Nevertheless, we think that this effect would still be independent of the concordance itself.

In pairwise comparisons, one caveat was that not all concordant stimuli differed significantly from all discordant stimuli. We showed that Flicking + Rattle concordant stimuli prompted significantly stronger responses than Waving + Rattle discordant stimuli, although not significantly stronger than Flicking + High-frequency discordant stimuli. We think, however, that this discrepancy in results does not contradict our conclusions. Although some of the post-hoc (a-posteriori) tests do not confirm our main hypothesis, none of them contradicts it. At the same time, our main hypothesis does not refer to any of these pairwise comparisons, but to the a-priori contrast between concordant and discordant stimuli—and for this general comparison we obtained results that were significant for each of the tested variables (Results). The discrepancies in the results of post-hoc tests may also suggest that our study was underpowered. We did not conduct an a-priori power analysis because we had no basis for predicting the effect size of concordance. Therefore, we relied on a rule of thumb based on the Central Limit Theorem. In experimental field ecology research, the rule is that a sample size of at least 30 is safe^28^, and we more than doubled that number. Our sample size should therefore be sufficient to obtain statistical significance for a scientifically significant effect, without overpowering the study and detecting trivial effects.

Our results imply that the study of how modalities are perceived and integrated is a necessary step to understanding the function of multimodal signals in general. Research on signalling in the sexual context is mostly unimodal. Where in turn, multimodal signalling is studied, the main emphasis is placed either on function^29,30^ or perceptual mechanisms^31–35^. However, understanding the function of a multimodal signal requires taking into account perceptual mechanisms, as indicated by a growing number of studies^36–39^. For example, by combining sound and motion in different structural and temporal configurations, we obtain not one multimodal signal but a whole set of signals. Depending on how the two modalities are combined, movement added to singing can enhance or weaken reactivity, or it can evoke completely different reactions^11,13,40–44^. The concordance effect we describe here is a consequence of multimodal perception, which means that it is the specific convolution of the components that is responsible for the response. The results of this and similar experiments therefore indicate that disregarding mechanisms of multimodal perception can lead to erroneous or false negative conclusions about the function of multimodal signals^25^.

The concordance of the starling movements and songs may provide the same basis for multimodal perception as the temporal and spatial congruence of components ^42^. In humans, music is usually used as the basis for improvisation by a dancer who imitates sounds with movement because music and movements share a common structure that affords equivalent emotional expressions^45^. This relationship is not limited to performance alone, as the structures responsible for musical and visual processing are cognitively related as well^46^. Some animal studies have also shown that body movements can be choreographically linked to simultaneous sounds^14,16,47^. At the same time, even in distantly related birds, brain systems that control vocal learning are directly adjacent to brain systems involved in movement control^48^, providing compelling support for a causal link between the capabilities for vocal imitation and dance. Starlings are open learners and great imitators of sounds of other species^49,50^. If specific movements are choreographically matched with specific song phrases, this might be a strong argument for their perceptual relationship.

## Materials and methods

### Study site and species

We studied starlings inhabiting Szczytnicki Park in Wrocław, Poland. This area of over 1 km^2^ is covered with a large number of old trees and lawns, providing a suitable habitat for starlings, which breed there at a high density^51^. After spring arrival, males start occupying nest holes and intensively display^20,23,52^. Male starlings sing in the presence of both females and males^20,23,52,53^. Although males are more likely to approach singing males than females^24,54^, they are rarely aggressive and often sing alongside each other^21,53,55^.

Starlings produce long and complex songs that typically include four categories of phrases (series of notes), carried out in a predictable sequence^19,56^. Songs usually start with whistles, i.e., loud, pure-tone sounds, spaced by approximately 1 s pauses^56^. Whistles are followed by variable phrases, which contain a variety of complex notes of relatively low amplitude and with only short temporal gaps in between. Many such notes cover a wide frequency range (1–8 kHz) in a short time, giving the impression of a series of clicks, buzzes and trills^27^. Next, rattle phrases are sung, which are characterized by a rapid series of clicks (so-called click trains) running through the phrase as other sounds are being produced. A song bout is completed by the relatively loudest high-frequency phrases (6–10 kHz)^27^.

**Figure 2:**
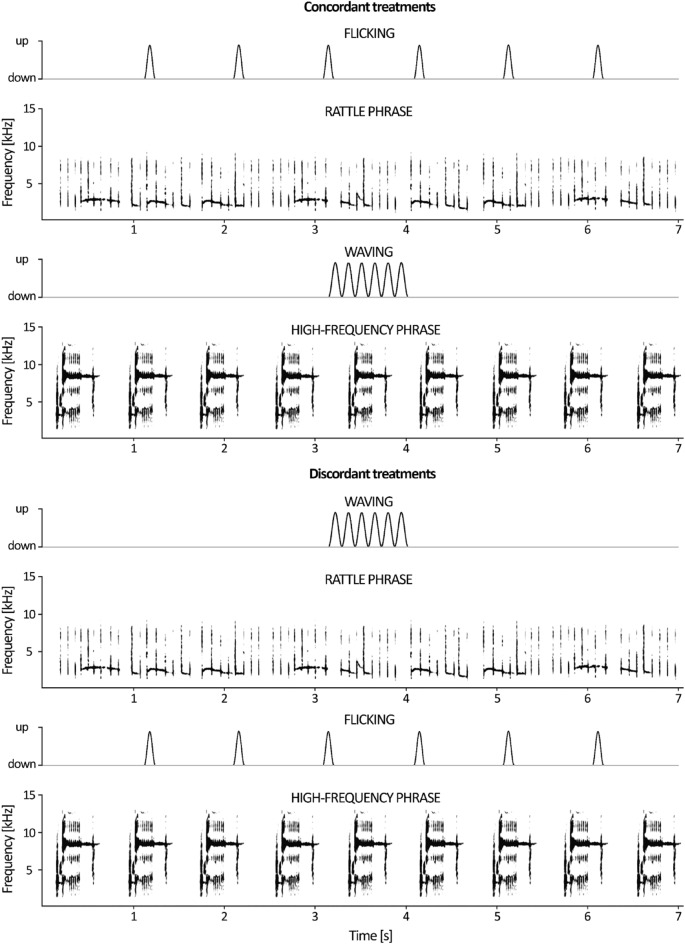
Combinations of visual and acoustic stimuli in treatments.

Male songs are often accompanied by two kinds of wing movements, which differ both in the range of motion and temporal organization. During wing waving, half open wings rotate around shoulders (Supplementary Videos 1), while during wing flicking, wings are slightly lifted in rapid flushes (Supplementary Videos 2)^19^. Earlier observations showed that movements accompany 60% of song bouts; flicking was observed in 73% and waving in 33% of song bouts accompanied by movements. At the same time, 86% of wing flicks were performed during rattle phrases of songs, and 68% of wing waves were performed during terminal high-frequency phrases^22^. Despite the structural match, the temporal structure of these songs and movements is far from compatible. While the flicks seem to be matched to the pulses of the song (if in a rattle phrase), the waves are sequences of movements that do not exactly match any phrase of the song (Fig. 2). Therefore, apart from the nonrandom juxtaposition of specific types of movement and song, the whole display is more like a song-based movement improvisation than a fixed temporal pattern (Supplementary Videos 1 & 2).

Wing-waving has also been reported to occur without song, but the frequency of this behaviour is unknown^19,22^. Observations of captive birds have suggested that wing movements occur primarily during courtship^22,23,57^. However, under natural conditions, it is easy to observe males moving in the presence of only other males or in the absence of any other starling.

### Experimental design

We tested a total of 74 birds to determine if the concordance of movements and songs affects the efficacy of their audio-visual signals. We used a scheme with independent groups in which each experimental male was assigned to one of four treatments consisting of audio-visual displays (Fig. 2). In each of the treatments, the birds were stimulated by audio-visual displays, consisting of the movement of the wings of the robotic model and the song played over a loudspeaker. In the concordant flicking treatment (n = 17), wing flicks were combined with the rattle song, whereas in the discordant flicking treatment (n = 20), flicks were accompanied by high-frequency terminal songs. Analogically, in the concordant waving treatment (n = 17), wing waves were accompanied by high-frequency terminal songs, whereas in the discordant waving treatment (n = 20), wing waves were accompanied by rattle songs.

To synthesize the acoustic stimuli, we used recordings from 20 males, different than the birds used in the experiment. From each individual, we selected four high-quality recordings, which were used in one series of four treatments. We used four recordings from each individual because the individual's songs showed much less structural variation of the phrases than the songs of different individuals. The recordings lasted 7–9 s, two of which consisted of 9–12 high-frequency terminal trills and another two consisted of the same number of rattle phrases. These ranges were due to differences between individuals. Therefore, instead of manipulating the length of phrases coming from different males, we preferred to use sequences of similar phrases that occur naturally in songs of one male without manipulation. Both types of songs were next combined with the two types of movements to create the four treatments. The recordings were made with the Sennheiser ME66 cardioid microphone coupled with the Olympus LS-10 solid-state recorder (sampling frequency 44.1 kHz, 16 bit) and edited using Avisoft-SASLab Pro (Avisoft Bioacoustics, Berlin, Germany).

### Robotic birds

We used a taxidermic robotic male starling during playback experiments. The construction was based on two digital servo-motors (KST Digital Standard Brushless Servo MS805) placed under the model bird and attached to the humeri of wings with separate transition shafts. Thanks to this mechanism, our robot could imitate the natural behaviour of starlings. Servo-motors and audio playback were controlled together by a circuit board based on the Arduino platform (Arduino Micro; www.arduino.cc). This enabled us to precisely program the timing of wing movements and vocalizations. The whole set was remote-controlled using a custom-designed radio controller based on the X-bee platform (Digi International, Minnetonka, MN, USA).

We used video recordings of natural displays to reproduce movements of the wings. To mimic wing-waving, we used the model bird with partially opened wings moving forth and back (Supplementary Video 3). In the middle of each song playback, one series of 6 wing beats (movement range: 45°, duration: 1.0 s each) was given by the model. In wing-flicking treatments, the wings of the robotic bird were folded and moved up and down in 6 rapid flushes (wing beats) that lasted approximately 0.14 s each (Supplementary Video 4). During each song playback, the model performed six wing flicks in one second intervals starting one second after the playback.

### Field procedures

Experiments were carried out between 23 April and 4 June 2020 in the morning hours (0600–1200 CEST). The order of treatments was randomized, and on each study day, we performed all 4 treatments with calls from one male. Before the treatment, the robotic model and the loudspeaker were mounted on the 7 m high stand to maximize its visibility. We placed the stand in the breeding sites of starlings, avoiding close proximity (less than 20 m) to nest holes. Song playbacks were broadcast from a JBL Charge 3 amplified loudspeaker (20 W, frequency range 65–20 000 Hz) at natural amplitudes of 60–65 dB SPL(A) at 10 m (measured from three individuals with UNI-T UT352 Sound Level Meter). Starlings are not territorial but social and curious, and they respond with positive phonotaxis to conspecific songs. Therefore, we lured the males near our model by playing conspicuous fragments of the starling male song and started the treatments as soon as a focal bird approached the model closer than 15 m. At the same time, we ensured that the bird had an unobstructed view of the taxidermic model. We only lured birds that were close to the given range. Therefore, the bird either immediately moved towards the model, and we started the treatment, or it ignored us, and we resigned. As a result, the luring itself had a small and standardized influence on the later reactions to the treatments. The treatment lasted one minute and included 5 randomly distributed repetitions of one song playback (7–9 s long) matched with wing movements. To avoid repeated testing of the same individuals, we accepted that the next treatment should start not earlier than 10 min after the previous treatment and at a distance of not less than 100 m. In practice, for greater certainty, we usually multiplied these values, taking into account, e.g., the direction from which the birds flew.

To compare the reactions of starlings to the treatments, we measured the time the birds were near the robot model (< 15 m), the shortest distance to the robot, and whether the birds were following the audio-visual stimulus. The time was measured from the beginning of the treatment to the moment the focal bird flew away from the model. The shortest distance to the robotic bird was initially estimated on the basis of observation and then confirmed using a laser rangefinder (Bushnell Yardage Pro). Following the stimulus refers to the movement of the bird during the treatment. After the bird had flown to less than 15 m, it either remained stationary (scored as 0) and showed no further interest in the stimulus, or it flew between the branches towards the model (scored as 1). We recorded treatments with a video camera set on a tripod 1.5 m above the ground, and we recorded audio notes in real time. Since we operated in a shaded area covered with trees, we relied primarily on direct observation and audio notes, using video recordings only for correction. We measured only males’ responses to the treatments because natural starling displays are known to attract mostly same-sex conspecifics^24,54^. We used the morphological features described in Kessel^58^ and Smith et al.^59^ to sex experimental birds.

### Statistical analysis

We used generalized linear mixed models (MIXED) to compare bird responses to different treatments. This procedure is suitable for clustered and nonnormally distributed data. In our experiment the data was clustered because to create 74 audio playbacks we used song recordings from 20 birds. We used songs from a single male during treatments with 4 focal males, which means that the responses of such 4 focal males were not independent, and the mixed models allow for this data structure to be taken into account. We first compared responses to all four treatments. We then nested the treatment effect within the concordance effect (two concordant treatments together vs. two discordant treatments together) to test whether differences between treatments were due to concordance or differences within both these categories. Additionally, we used post hoc Fisher’s LSD method to create confidence intervals and compare the means of all treatments. We separately analysed three measures of response: time, minimal distance, and following the stimulus. We fitted the models using Gaussian error distribution with logarithmic link function for time and distance variables and binomial error distribution with complementary-log–log link function for the following the stimulus variable. The identity of the bird whose song recordings were used to create the audio playbacks was used as a random factor in the models. Except for the following the stimulus variable, this random effect was significant in every analysis. This therefore confirms that the use of recordings from one individual in four different treatments was justified. For all the analyses, we used IBM SPSS Statistics v. 27.0 (Armonk, NY, USA). All *P* values were two-tailed.

### Ethics statement

The experimental protocol adhered to the Animal Behaviour Society guidelines for the use of animals in research. Necessary permits were obtained from the Polish Regional Direction of Environmental Protection (WPN-II.6401.377.2017. AC, WPN.6401.345.2017.MK).

### Supplementary Information

Dataset S1.

Supplementary Legends.

Supplementary Video 1.

Supplementary Video 2.

Supplementary Video 3.

Supplementary Video 4.

## Data Availability

The data reported in this paper are available in electronic supplementary material, Dataset S1.
