# Supplementary material for: Concordance of movements and songs enhances receiver responses to multimodal display in the starling
Source: Sci Rep. 2024 Feb 13;14:3603. doi: 10.1038/s41598-024-54024-w (PMC10864327; doi:10.1038/s41598-024-54024-w)
Supplement: Supplementary file 2 — Supplementary Legends. [file 41598_2024_54024_MOESM2_ESM.docx]

**Supplementary Video 1 – Natural wing waving display.**

During wing waving half open wings rotate around shoulders,

**Supplementary Video 2 – Natural wing flicking display.**

During wing flicking wings are slightly lifted in rapid flushes

**Supplementary Video 3 – Imitated wing waving display**

Taxidermic robotic bird with partially open wings that move continuously and with a large range.

**Supplementary Video 4 – Imitated wing flicking display.**

Taxidermic robotic bird with folded wings that move intermittently and with a small range.
